# Recognition of DNA Termini by the C-Terminal Region of the Ku80 and the DNA-Dependent Protein Kinase Catalytic Subunit

**DOI:** 10.1371/journal.pone.0127321

**Published:** 2015-05-15

**Authors:** Derek S. Woods, Catherine R. Sears, John J. Turchi

**Affiliations:** 1 Department of Biochemistry and Molecular Biology, Indiana University School of Medicine, Indianapolis, Indiana, United States of America; 2 Department of Medicine, Indiana University School of Medicine, Indianapolis, Indiana, United States of America; University of South Alabama Mitchell Cancer Institute, UNITED STATES

## Abstract

DNA double strand breaks (DSBs) can be generated by endogenous cellular processes or exogenous agents in mammalian cells. These breaks are highly variable with respect to DNA sequence and structure and all are recognized in some context by the DNA-dependent protein kinase (DNA-PK). DNA-PK is a critical component necessary for the recognition and repair of DSBs via non-homologous end joining (NHEJ). Previously studies have shown that DNA-PK responds differentially to variations in DSB structure, but how DNA-PK senses differences in DNA substrate sequence and structure is unknown. Here we explore the enzymatic mechanisms by which DNA-PK is activated by various DNA substrates and provide evidence that the DNA-PK is differentially activated by DNA structural variations as a function of the C-terminal region of Ku80. Discrimination based on terminal DNA sequence variations, on the other hand, is independent of the Ku80 C-terminal interactions and likely results exclusively from DNA-dependent protein kinase catalytic subunit interactions with the DNA. We also show that sequence differences in DNA termini can drastically influence DNA repair through altered DNA-PK activation. These results indicate that even subtle differences in DNA substrates influence DNA-PK activation and ultimately the efficiency of DSB repair.

## Introduction

Efficient repair of DNA DSBs requires the recognition and resolution of a wide variety of DNA termini including variations in structure, DNA sequence, and chemistry. The DNA-dependent protein kinase (DNA-PK) is a heterotrimer consisting of Ku70, Ku80, and the catalytic subunit (DNA-PKcs). DNA-PK initiates the repair of the preponderance of DSB in mammalian cells and regulates the Non-Homologous End Joining Pathway (NHEJ) through its kinase activity. DNA-PK activation requires both protein/protein interactions within the heterotrimer and protein/DNA interactions, which are not mutually exclusive [1,2]. Following activation, DNA-PK phosphorylates several important targets including p53, RPA, and histone H2AX [3–8]. We have previously reported that the DNA to which DNA-PK is bound modulates DNA-PK activation [2,9]. Each strand of the DNA has a unique influence on DNA-PK activation as the 5’ end directly activates the kinase while the 3’ strand anneals DNA termini across the break. However, the mechanism by which DNA-PK recognizes different DNA substrates and responds to them specifically remains unknown [2]. Other studies have implicated a role for the C-terminal region of Ku80 in stimulating DNA-PK activity through protein/protein interactions; however, the location and extent of these stimulatory interactions is debated [10–12]. Multiple reports, including our own, have indicated that the C-terminal region of Ku80 undergoes large conformational changes upon the Ku heterodimer-DNA interaction and that the C-terminus of Ku80 directly interacts with DNA-PKcs [13–15].

We have tested the role of different structural regions within the C-terminus of Ku80 in activating DNA-PK as a function of DNA termini structure, length and sequence. Previous *in vivo* studies have provided limited mechanistic information regarding DNA-PK activation by DNA because mixtures of DNA substrates were used [10,11]. To address this issue, a series of *in vitro* assays were utilized to assess how the different Ku80 structural domains impact the enzymatic activity of DNA-PK with a level of resolution and sensitivity not currently possible with *in vivo* systems. The *in vivo* analyses, while limited in some terms, importantly allow the manipulation of DNA—PK activation in the context chromatin which has been postulated to influence NHEJ efficiency [16]. Our *in vitro* results demonstrate that distinct regions within the C-terminus of Ku80 are responsible for stimulating DNA-PK activity depending on the structure of the DNA substrate to which Ku is bound. This suggests that Ku recognizes different DNA structures based on DNA length, the presence of overhangs, and orientation of overhangs and subsequently differentially activates DNA-PK. Additionally, we demonstrate that the sequence of DNA substrates on 5’ overhangs can modulate DNA-PK activity. Interestingly, this modulation is independent of the Ku80 C-terminal structural regions suggesting that the discrimination is intrinsic to DNA-PKcs. Furthermore, the terminal sequence bias of pyrimidines versus purines in DNA-PK activation correlated with increased DNA repair via NHEJ *in vivo*.

Overall this study answers long held questions in the field of DSB repair and defines a novel mechanism by which DNA-PK recognizes distinct DNA termini. Differential activation of DNA-PK due to alterations in DNA structures is assigned to various interactions occurring between the protein structural regions within the C-terminus of Ku80 and DNA-PKcs, while differential activation due to terminal sequences are assigned to DNA/DNA-PKcs interactions. In addition, our data provide a mechanism for the conflicting reports on the role of Ku80 C-terminus in DNA-PK activation where Ku80 C-terminal truncation mutants either displayed no DNA-PK activation or nearly wild type activation. Our data demonstrate that these differences are largely a function of the individual DNA substrates employed in the different systems used to assess activity.

## Materials and Methods

### Expression and purification of Ku and DNA-PKcs

**Fig 1:**
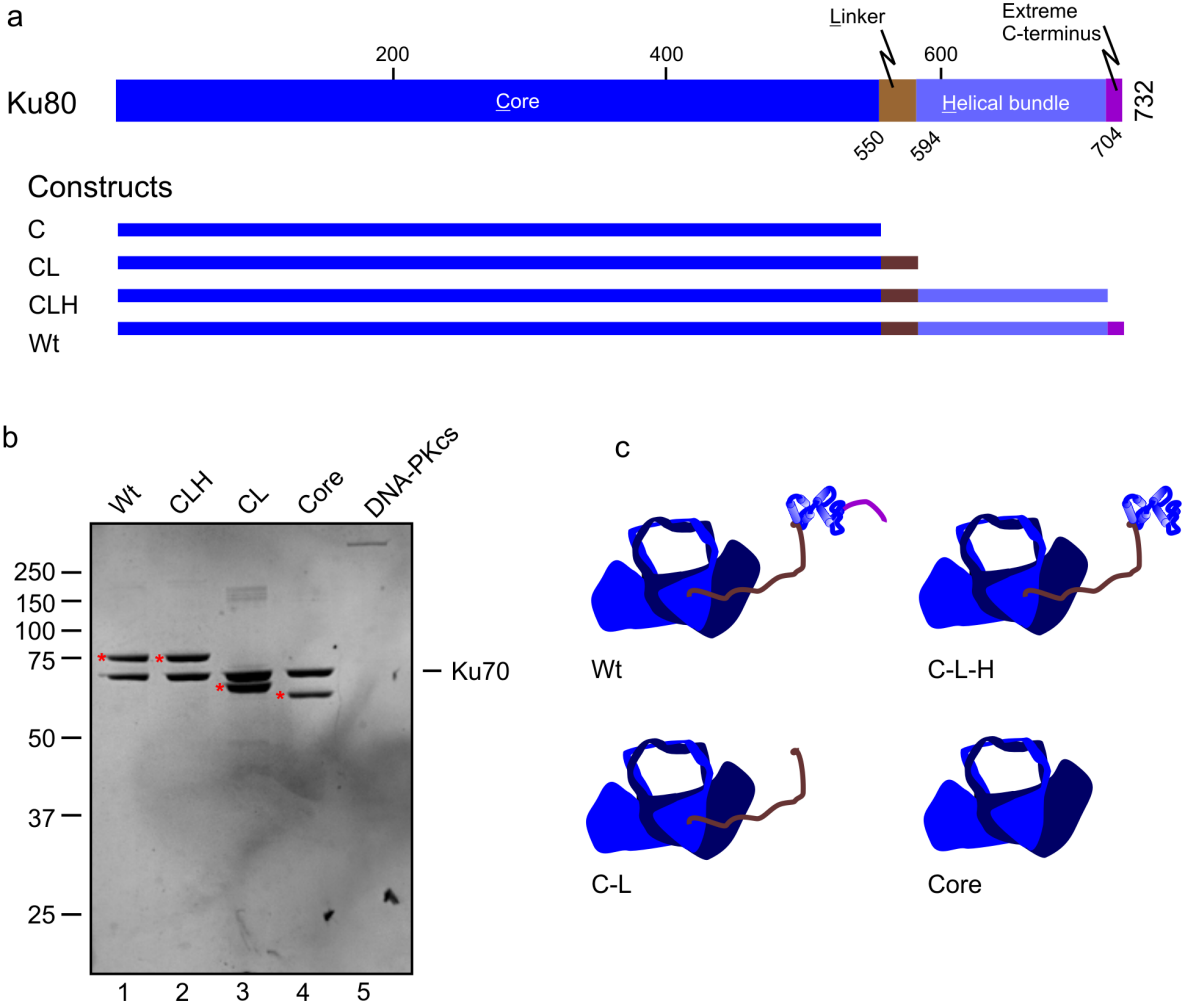
Ku80 Protein Structure, Mutant Construction, and Protein Purification. a) Linear representation of Ku80 structural domains and mutant construction. b) Coomassie stained SDS gel of purified Ku and DNA-PKcs proteins used in this study. All Ku80 mutants were purified with wild type Ku70 which remains consistent in lane 1–4. Ku80 mutant constructs are indicated along the top of the gel and the Ku80 protein bands are denoted with red asterisks. c) Model of wild type and Ku mutant constructs used in this study. Ku80 is depicted in dark blue and Ku70 is depicted in grey. The structural regions of the Ku80 C-terminus are depicted in their corresponding color from (a).

Ku80 truncation mutant constructs were created via site directed mutagenesis by inserting stop codons following the indicated amino acids and recombinant baculoviruses prepared (Fig 1A). The various Ku80 constructs were each co-expressed with wild type His-tagged Ku70 and purified from SF9 cells as previously reported by Ni-NTA affinity chromatography [9]. The Ni-purified Ku70/80 was further fractionated on a Q-Sepharose column and Ku70/80 containing fractions pooled, dialyzed and stored at -80°C until use. [14]. All mutant constructs were assessed for DNA binding activity by EMSA. DNA-PKcs was purified from HeLa cells as previously described [9,17]. Notably it was imperative that DNA-PKcs be purified in the absence of endogenous Ku and thus this was monitored throughout the purification via western blot. Importantly no detectible Ku70 or Ku80 was observed in final pools of DNA-PKcs (Fig 1B Lane 5) and kinase activity was stimulated at least 4-fold by the addition of purified Ku, depending on the specific DNA assessed. Final pools of all proteins were dialyzed in a dialysis buffer consisting of 20 mM HEPES pH 7.5, 75 mM KCl, 10% glycerol, 0.005% Triton X-100, and 2 mM DTT.

### DNA-PK Kinase Assays

Kinase assays were essentially performed as we have previously reported using the DNA and protein concentrations indicated [2,9]. Reactions using 30bp and 60bp substrates were performed with 500fmol of DNA per reaction while reactions with ~400bp and plasmid DNA were performed with 154fmol of DNA. Single stranded oligonucleotides were purchased from Integrated DNA Technology (IDT, Coralville, IA, USA) (S1 Table). The ~400bp substrates were purchased as double stranded “gBlocks” Gene Fragments from IDT(S2 Table). G50 spin-columns were used to purify the 397bp digested DNA from the short terminal fragments. Kinase assays using plasmid DNA substrates were performed with pcDNA3.1 digested with either XhoI, BamHI, EcoRV, and KpnI or pCAG-GFP digested with XbaI or EcoRI (New England Biolabs). The specific sequences recognized by the restriction enzymes and DNA termini generated are shown in S3 Table. Complete digestion of the plasmid DNA was analyzed by native agarose gel electrophoresis of the reaction products.

We and others have previously reported that the C-terminus of Ku80 is dispensable for Ku/DNA binding [17,18]. In order to control for slight variations in binding activity which may occur between protein purifications, equal amounts of active Ku as measured by DNA binding activity was used in kinase assays containing 42ng of DNA-PKcs. Titration experiments were performed and demonstrate that these concentrations of Ku and DNA were in excess and DNA-PKcs is the limiting factor for all enzymatic analyses (S1 Fig).

### DNA-PK Recruitment Assay

DNA-PK Recruitment was determined using an enzyme-linked immunosorbent assay (ELISA) as we have described previously [19,20]. Briefly, 2pmol of 5’ biotinylated 30mer top annealed to 30mer bottom, 60mer top annealed to 60mer bottom, and 421mer top annealed to 421mer bottom DNA was bound to streptavidin coated wells of a 96 well plate overnight at 4°C in 100μl. Unbound DNA was removed and wells were then blocked with 200μl of 2% BSA in TBS/Tween for 1 hour at room temperature. Wild type or Ku mutants (1000 units) were added to the DNA coated wells with 424ng of DNA-PKcs in a total volume of 100μl of dialysis buffer. Protein complexes were allowed to bind DNA at room temperature for 1 hour. The unbound protein was removed and again wells were blocked with 200μl 2% BSA in Tris-buffered saline/ 0.5% Tween (TBS/Tween) for 1 hour at room temperature to avoid non-specific antibody binding. 100μl 2% BSA in TBS/Tween and a 1:750 diluted primary antibody specific for DNA-PKcs (Calbiochem) was added to each well and incubated at room temperature for 1 hour. Then wells were washed with 200μl of 2% BSA in TBS/Tween three times for 5 minutes each. 100μl of 1:2500 diluted goat anti-rabbit HRP secondary antibody in 2% BSA in TBS/Tween was added to each well and incubated for 1 hour at room temperature. Wells were washed three times with 200μl of 2% BSA in TBS/Tween for five minutes in each wash. 100μl of 1-Step TMB ELISA reagent (Thermo Scientific) was added to each well and a kinetic read of absorbance 370 nm was initiated at 37°C and read every 5 minutes for 60 minutes total. The average change in optical density at 370 nm per minute is reported at 15 minutes where the reaction remained linear with time. Each condition is reported as an average of triplicate determinations and error bars indicate one standard deviation. Standard curves were used to evaluate the responsiveness of the assay (data not shown).

### Protein DNA interactions analysis, electrophoretic mobility shift assay

Electrophoretic Mobility Shift Assays (EMSAs) were used to evaluate DNA binding activity of Ku on 30bp and 60bp blunt ended DNA substrates as we have previously described [2,14,21]. All Ku preparations, mutant and wild type, were assessed for DNA binding activity to allow normalization between the various constructs and protein preparations. Reactions were performed in a volume of 20 μl containing 500 fmol of DNA (30bp blunt substrate). Gels were run using standard conditions and were visualized using Phosphoimager analysis. One unit of Ku DNA binding activity is defined as the amount of Ku necessary to bind 50 fmol of DNA in an EMSA containing 500 fmol of DNA.

### Cellular NHEJ/ Host Cell Reactivation Assay

Linearized pCAG-GFP was used in a host cell reactivation assay as pervious reported in H460 cells with only minor modifications [22]. The NHEJ reporter plasmid was linearized by indicated restriction enzyme digestion as recommended by manufacturer (New England Biolabs). Covalently closed circular pCAG-Red was used as a transfection control in all experiments. All transfections were performed using 2ug of the indicated plasmid DNA. 48-hours following transfection, cells were fixed on the coverslip, counterstained with DAPI and fluorescent images captured. The independent red and green fluorescence images were pseudo colored using ImageJ and an overlay figure prepared merging the two images as previously reported [22]. Quantification was performed scoring a minimum of 100 cells in each condition. Results reported are the average and SD of 6 independent transfections.

### Statistical Analysis

All statistical analysis was completed using Sigma Plot 12.0 software using a Student’s t- test. The P<0.05 was used as a significance threshold for all tests unless otherwise indicated. All statistical analysis of kinase assays and ELISAs were from triplicate determinations.

## Results

### Ku Mutant Design and Purification

To determine if the Ku80 C-terminus plays a role in DNA discrimination by DNA-PK, a series of Ku80 C-terminal truncation constructs were constructed based on the Ku 80 structural features and domains **(** Fig 1A). The first construct was engineered with a stop codon after core DNA binding/Ku70 dimerization domain (amino acid 550) to create the “core” construct. A stop codon engineered after the disordered Linker domain (amino acid 594) resulted in the core-linker construct (C-L). A stop codon placed after the α-Helical bundle (amino acid 704) to create the core-linker-helix (C-L-H) construct. Each of these constructs was compared to the full length, wild type construct which contained the core-helix-linker and extreme C-terminus. These constructs were each used to generate recombinant baculoviruses and were co-expressed with wild type Ku70 and purified from infected SF9 insect cells using a two—column procedure to ensure a high degree of purity and without any contamination DNA-binding activity. A coomassie blue stained SDS gel of the final pool of protein from each construct is presented in Fig 1b (lanes 1–4). DNA-PKcs was purified from HeLa cells as previously reported [17] **(** Fig 1b, lane 5) and was devoid of endogenous Ku as assessed by western blot analysis of the DNA-PKcs pool of protein using (data not shown). A cartoon model of the Ku constructs is shown in Fig 1c.

### Different Structural Regions of the Ku80 C-terminus Stimulate DNA-PKcs Activity Depending on the Structure of the DNA Substrate

**Fig 2:**
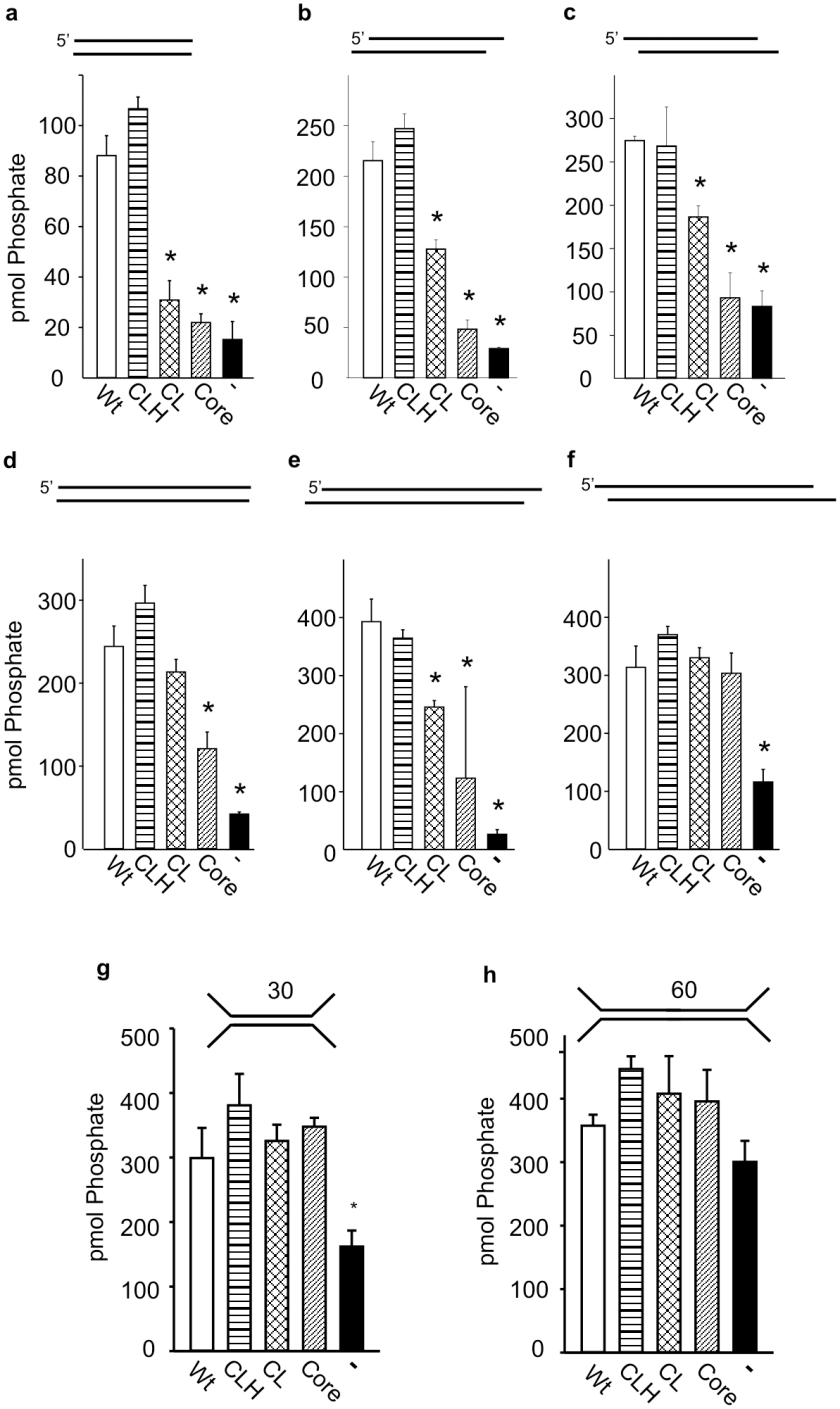
Different Structural Regions of the Ku80 C-terminus Stimulate DNA-PKcs Activity Depending on the Structure of the DNA Substrate. DNA-PK kinase activity is reported as pmol of phosphate transferred using the indicated DNA substrates. Reactions were completed with 30bp substrates containing a blunt (a) 3’ overhang (b) or 5’ overhang (c). DNA-PK activity was assessed on 60 bp substrates with blunt (d) 3’ or 5’ (e and f respectively. Y-substrates of 30 and 60bp are presented in panels g and h respectively. Ku constructs used in the reactions are indicated along bottom of the figure. Wild type bars are white, CLH bars contain horizontal lines, CL bars contain crosshatching, Core bars contain diagonal lines and no added Ku (-) bars are black. The 5’ and 3’ overhangs were prepared by digestion of the Blunt-ended gBlock with EcoRI and KpnI respectively. Sequences and other details concerning DNA substrates can be found in S1, S2, and S3 Tables. Data are presented as the mean and SD with asterisks indicating statistically significant differences compared to wild type (p <0.05).

We have previously reported that DNA-PK activation is influenced by the structure of the DNA substrate to which the holoenzyme is bound [2,9]. We interrogated how the different structural regions within the C-terminus of Ku80 impacted activation of DNA-PK as a function of DNA substrate structure and length (Fig 2). The sequence of the DNA substrates used is presented in S1 Table. Analysis of DNA-PK activation with the Ku80 constructs on a 30bp fully duplex DNA in presented in Fig 2A. No reduction in activity was observed upon deletion of the extreme C-terminus of Ku 80 (CLH construct) compared to the wild type. Interestingly, removal of the helical bundle (CL construct) resulted in a significant decrease in activity, which was also observed in the core construct which was devoid of each the linker, as well as the helix and extreme C-terminus. These data demonstrate the requirement for the α-helical domain for full DNA-PK activation (Fig 2a). A modest reduction in activity was observed in the core construct compared to the CL construct though neither of these were significantly greater than the no Ku control. Analysis of 30bp substrates with either 4 base 5’ or 3’ overhangs (Fig 2b and 2c) showed somewhat different results. While the CLH mutant, devoid of only the extreme C-terminus retained full wild type kinase activity, the CL mutant containing the devoid of both the helix and extreme C-terminus also had kinase activity above the no Ku control, albeit at ~50% of the wild type level of activation. In contrast, this CL mutant did not stimulate DNA-PKcs activity above background on the 30bp blunt ended substrate (Fig 2a). These data suggest that the single stranded overhangs (both 3’ and 5’) can partially compensate for helical bundle in the activation of the kinase.

Analysis of longer DNA substrates, 60bp in length, revealed kinase activation that, similar to the 30bp substrate, was independent of the extreme C-terminus. The activation achieved on these longer substrates, however did reveal differences as a function of the Ku80 construct used. An intermediate level of stimulation was observed in reactions with the Ku80 core mutant on the blunt and 3’ overhang (Fig 2d and 2e) while full, wild type levels of activation were observed with the 60bp 5’overhang substrates (Fig 2f). Further, the level of activation by the CL mutant, containing the disordered linker region, was statistically indistinguishable from wild type (p = 0.139) in the presence of 60bp blunt-ended and 4 base 5’ overhang substrates while only intermediate activation was observed with this mutant in reactions performed with the 60bp substrate with 4 base 3’ overhangs (Fig 2d–2f). Together these data indicate that the helical bundle and disordered linker region are necessary for wild type levels of DNA-PK stimulation when the complex is bound to certain DNA structures while being dispensable on others. To further interrogate the influence of terminal overhangs on Ku80 dependent DNA-PK stimulation, Y- substrates were utilized to essentially provide single strand overhangs on both the 5’ and 3’ termini. In the presence of the 30bp Y DNA substrate, Ku stimulates the kinase to wild type levels even in the absence the Ku80 C-terminus similar to the results observed with the 60bp 5’ overhang substrate (Fig 2g and 2h). Interestingly, in the presence of the 60bp Y substrate, Ku is completely dispensable for maximum activation of DNA-PK no statistically significant difference was observed between the no Ku and wild type controls (Fig 2h).

**Fig 3:**
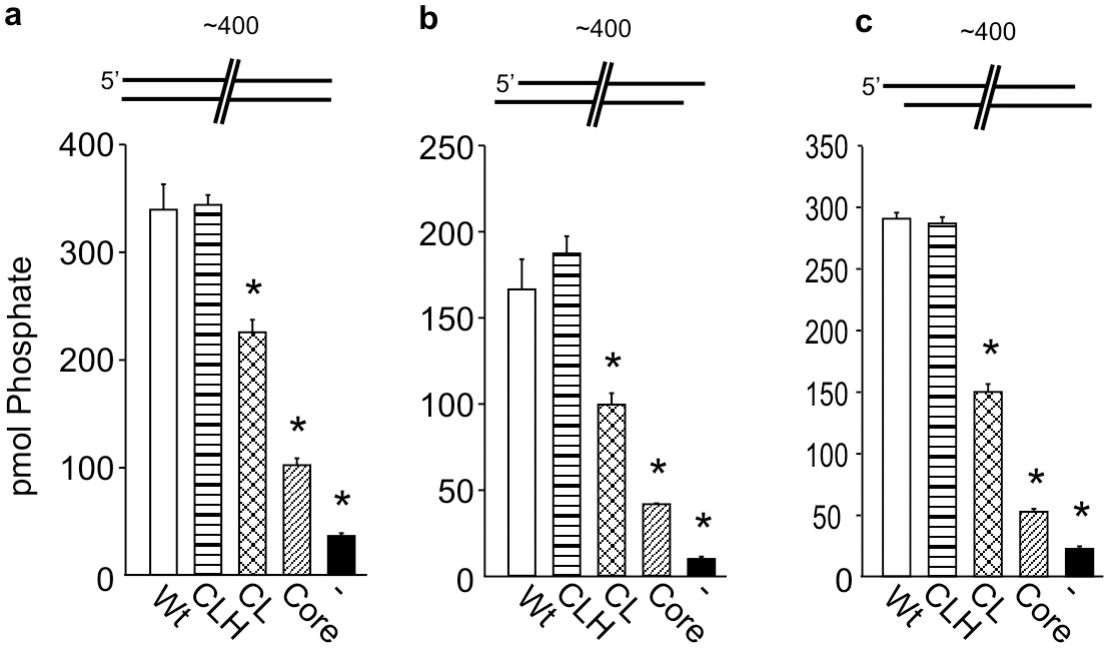
DNA-PK activity stimulated by 400bp substrates. DNA-PK kinase activity is reported as pmol of phosphate transferred using the indicated DNA substrates. Reactions were completed with 400bp substrates containing a blunt (a) 3’ overhang (b) or 5’ overhang (c). Sequences and other details concerning DNA substrates can be found in S1, S2, and S3 Tables. Data are presented as the mean and SD with asterisks indicating statistically significant differences compared to wild type (p <0.05).

The observed differences in activation between the 30bp substrates and the 60bp substrates even when the terminal structures were the same were attributed to length of the DNA which could influence complex formation. Accordingly, we tested the influence of significantly longer DNA substrates on these activation relationships. Overall the affect or the Ku80 C-terminus on DNA-PK activation was similar in reactions performed with blunt ended and 3’ overhang ~400bp substrates as their respective 60bp counterparts (Fig 3a, 3b and 3c). The only major difference among these substrates is that the CL mutant is unable to restore wild type levels of activation on the ~400bp blunt end unlike the blunt end 60bp DNA. The CLH mutant was able to fully activate the kinase to wild type levels while the CL mutant was able to partially stimulate the kinase. Additionally the Core mutant, which completely lacks the C-terminal region, can stimulate the kinase above background and these differences are statistically significant. In contrast, the ~400bp substrate containing 4 base 5’ overhangs alters the influence of the C-terminus on DNA-PK activation in such a way that is similar to the 30bp substrates containing 4 base 5’ overhangs (compare Fig 3c to 2c and 2f). Here the CLH mutant is sufficient to activate DNA-PKcs to wild type levels, with partial activation using the CL mutant and above background activation using the Core mutant (Fig 3c). These activation relationships are summarized in Table 1 and together illustrate the importance of C-terminus of Ku80 on DNA-PKcs activation as distinct structurally defined protein regions differentially influence kinase activation depending of the structure of the DNA substrate to which they are bound.

**Table 1:** DNA-PK activation.

DNA substrate		Ku Constructs*			
Length	Term. struc.	wild type	C-L-H	C-L	Core
30bp	Blunt	++++	++++	-	-
	5'	++++	++++	++	-
	3'	++++	++++	++	-
	Y	++++	++++	++++	++++
60bp	Blunt	++++	++++	+++	++
	5'	++++	++++	++++	++++
	3'	++++	++++	++	++
	Y	Ku Independent			
~400bp	Blunt	++++	++++	++	+
	5'	++++	++++	++	+
	3'	++++	++++	++	+
Plasmid	Blunt	++++	+++	++	-
	5'	++++	++++	++	-
	3'	++++	++++	++	-

*Each plus sign indicates 25% of kinase activity for each DNA substrate normalized to wild type activity levels.

### The Ku80 C-terminus is Dispensable for DNA-PKcs-DNA Binding

**Fig 4:**
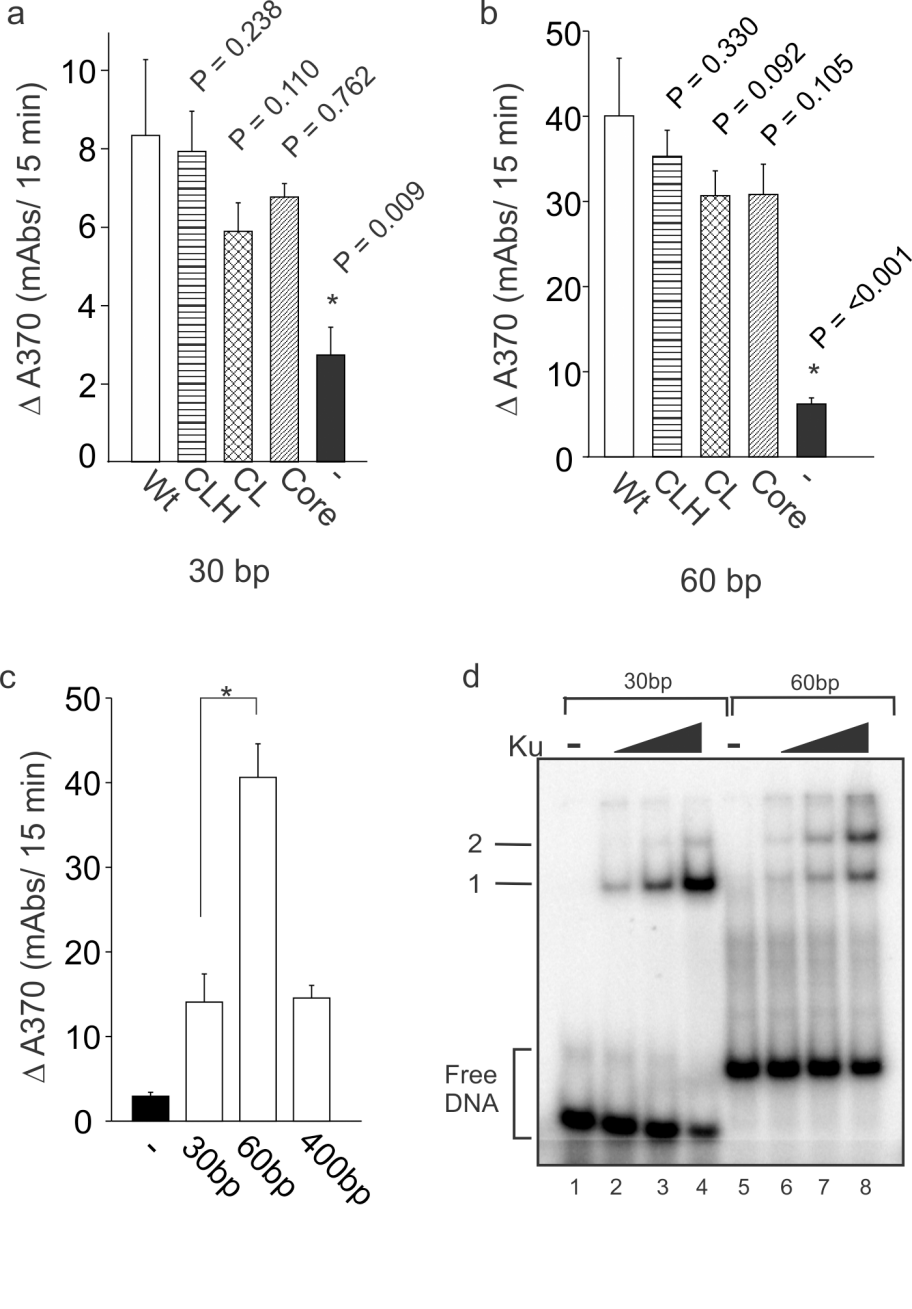
The Ku80 C-terminus is Dispensable for DNA-PKcs-DNA Binding. A modified ELISA was used to monitor DNA-PKcs-DNA binding and was performed as described in Materials and Methods. (a) Recruitment on 30bp DNA (b) Recruitment on a 60bp DNA. (c) DNA substrate influence on DNA-PKcs-DNA binding was tested on indicated substrates. Data are reported as the mean and SD of the rate of change in absorbance at OD_370nm_ in 15 minutes. (a-b) Asterisks indicate statistically significant differences compared to wild type (p<0.05). (c) Differences between 60bp vs 30bp substrate were statistically significant (p<0.05). (d), DNA binding Activity of Ku on 30bp and 60bp DNA. EMSA analysis of DNA binding activity was performed as described in Materials and methods. Ku was titrated at equal concentrations with the 30bp DNA (Lanes 1–4 and 60bp DNA (lanes 5–8). Products separated via native electrophoresis and imaged on a PhosphorImager. The free DNA is indicated by the bracket, and protein-DNA complexes representing a single Ku bound to a DNA and 2 Ku molecules bound to a DNA are indicated by the arrows.

Differences in DNA-PK kinase activity as a function of DNA co-factor length or structure could result from effects on DNA binding, effects in the catalytic cycle or both. The DNA binding activity of the various Ku70/Ku80 constructs used in this study were not statistically different from the wild type full length construct, consistent with other reports using different Ku80 mutants [23]. However, the role of the Ku80 C-terminal region, if any, in supporting DNA-PKcs to a DNA terminus and site of a DSB has been debated. While some evidence shows that the extreme C-terminus of Ku80 is necessary and sufficient for DNA-PKcs to bind to DNA [12], others show that the C-terminus has only a modest impact on DNA-PKcs-DNA binding [10,11]. Accordingly we asked if the differences we observed in kinase activation as a function of DNA length were the result of alterations in DNA-PKcs-DNA binding activity. Therefore, an ELISA based interactions assay was employed where the DNA to which DNA-PK binding was assessed was biotinylated on one end and bound to the wells of a streptavidin coated 96-well plate. The bound DNA was then incubated with the indicated Ku construct and purified DNA-PKcs. Unbound protein was washed away and DNA-PK-DNA complexes were detected using a DNA-PKcs specific antibody. Results using the 30bp (Fig 4A) and 60bp substrates (Fig 4b) illustrate a greater than 4-fold increase in DNA-PKcs with wild type Ku consistent with the DNA dependence of the PKcs binding event [24,25]. Each of the mutants and wild type Ku were able to effectively recruit DNA-PKcs to DNA, but no statistically relevant differences were observed between the mutants and wild type. Taken into consideration with the results of the kinase assay that the CL and Core mutants did not stimulate DNA-PKcs above background in the presence of this substrate (Fig 2a), these data suggest the differential DNA-PKcs stimulation result from differences in catalysis and not from differences in DNA-PKcs-DNA binding efficiency. Further the 30bp duplex is the most restrictive substrate in terms of lateral movement of the protein complex on DNA which in turn would also be expected to lead to the highest level of molecular crowding events. Such events would cause the most restriction to DNA-PK-DNA complex formation and retention of the complex on DNA. Despite this, the Core mutant was able to recruit and retain DNA-PKcs on the 30 base duplex DNA at levels that were statistically indistinguishable from wild type. Similarly, the Core mutant was able to recruit and retain DNA-PKcs at wild type levels on the 60bp and 400bp substrates (Fig 4B and Data not shown). These data provide strong evidence that the entire C-terminus of Ku80 is dispensable for Ku-dependent DNA-PKcs-DNA binding.

The results in Fig 2 indicate profound differences in DNA-PK stimulation by the C-terminus of Ku80 among different DNA substrates, with the most dramatic differences observed with the 60bp substrates. We tested if DNA-PK complexes form more readily on these different substrates. 60bp substrates recruit and retain more than double the amount of DNA-PK compared to 30bp and 400bp substrates which themselves were statistically indistinguishable (Fig 4C). This is an interesting result considering it is not obvious why a longer DNA substrate would result in reduced DNA-PK/DNA complex formation. It is possible that increased DNA-PKcs recruitment on the 60bp substrate negates the stimulatory effect of the Ku80 C-terminus. Ku-DNA binding on 30bp and 60bp substrates was assessed using EMSA assays. Results indicate that among equal concentrations of protein and DNA, Ku primarily binds 30bp DNA in a 1:1 ratio. Interestingly, the 60bp DNA is bound by both 2 molecules of Ku and 1 molecule of Ku with preference for the multiple binding events (Fig 4D). This is consistent with a previously described phenomenon which showed that cooperative Ku loading on DNA substrates ends is length dependent [26]. Of the DNA substrates evaluated for their ability to stimulate DNA-PK, the 60bp substrates show the greatest divergence in relation to the effect of the Ku80 C-terminal regions. The 60bp substrates are also unique in that they can accommodate 2 Ku molecules, but unlike the longer 400bp substrates, both Ku molecules are in close proximity to DNA termini where DNA-PKcs is known to bind [27], which may be responsible for this interesting distinction.

### Distinct Influences of the Ku80 C-terminus on DNA-PK Activation using Linearized Plasmid DNA

**Fig 5:**
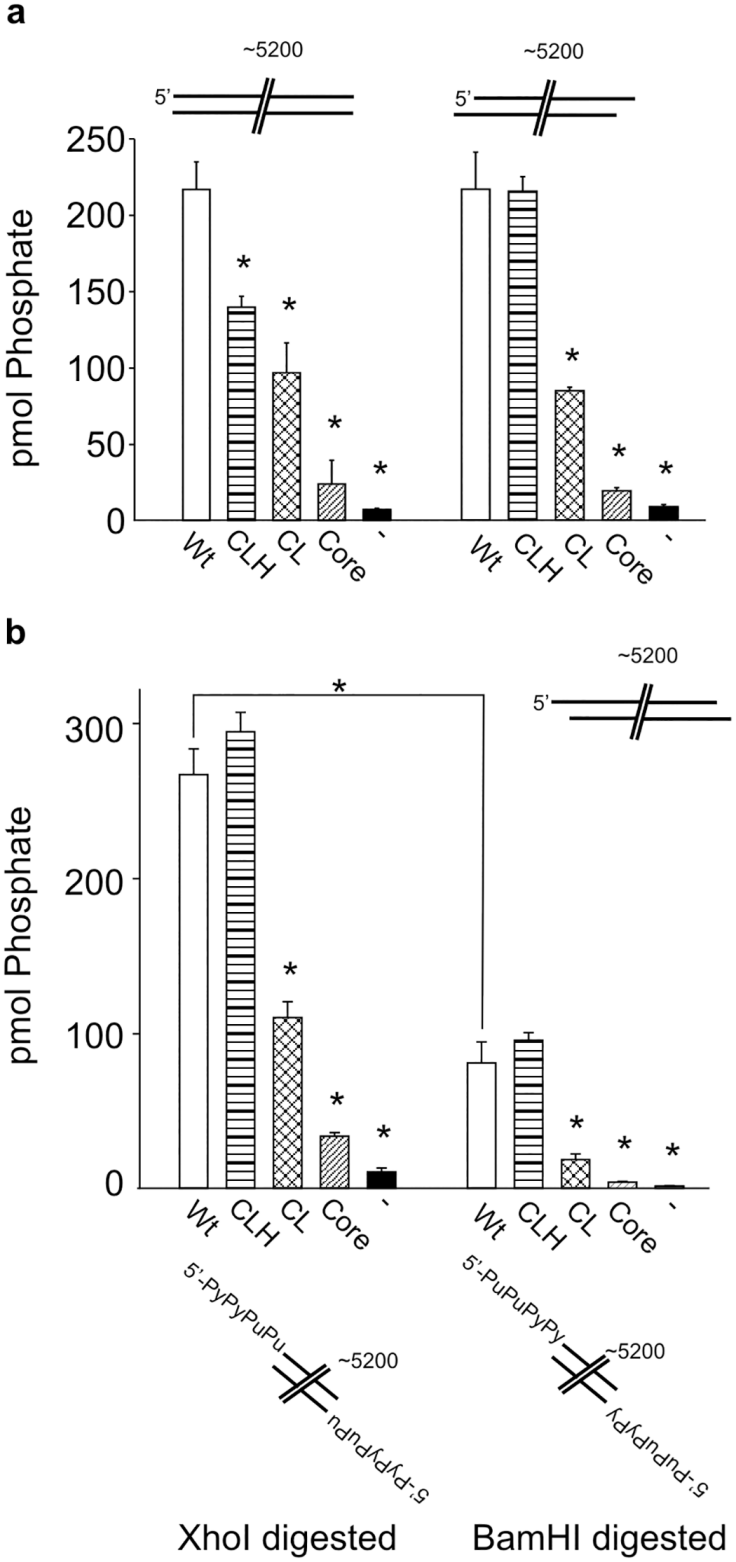
Distinct Influences of the Ku80 C-terminus on DNA-PK Activation with Linearized Plasmid DNA. a) DNA-PK kinase stimulation with plasmid DNA linearized with EcoRV generating blunt-ended termini and with KpnI generating 4 base 3’ single stranded overhangs. b) DNA-PK kinase stimulation with plasmid DNA linearized with XhoI and BamHI generating 4 base 5’ single stranded overhangs. DNA substrates are depicted pictorially. DNA termini generated by digestion are depicted below each graph indicating locations of pyrimidines (Py) and purines (Pu). Kinase activity is reported as the mean and SD of pmol of phosphate transferred. Asterisks indicate statistically significant differences compared to wild type (p <0.05). (b) Asterisk comparing wild type of XhoI digested DNA and wild type of BamHI digested DNA indicates statistically significant differences (p<0.05).

Substantial differences in DNA-PK activation were observed between substrates of various lengths (Fig 2). In order to evaluate the longest DNA substrates available while maintaining a homogenous preparation of substrates, plasmid DNA was linearized with various restriction enzymes yielding blunt ends, 4 base 3’ overhangs and 4 base 5’ overhangs and used in kinase assays as before (S1 Table). Additionally, using these substrates to test DNA-PKcs activation was advantageous because it is possible to assess their DNA repair *in vivo*. Analysis of activation on the plasmid DNA led to a series of interesting results and interactions. The blunt ended plasmid DNA for the first time revealed a role for the extreme C-terminus as the activation with the CLH was significantly reduced compared to wild type Ku **(** Fig 5a), an effect not observed with the 30, 60 or 400bp DNA substrates. This result is consistent with the extreme C-terminus harboring the previously defined “PIKK interaction motif” which has been reported to be necessary and sufficient for Ku80/DNA-PKcs interactions [12,28], but is in stark contrast our enzymology data demonstrating Ku dependent activation in the absence of the extreme C-terminus on 30,60 and 400bp substrates. Interestingly, the reliance on the extreme C-terminus for kinase activation was lost on both plasmid DNA substrates containing 5’ or 3’ overhangs where the CLH construct displayed reduced kinase activity compared to wild type (Fig 5a and 5b). Further loss of activity was observed in reactions containing the CL indicating that the α-helical domain was also involved in supporting kinase activation. Finally the core mutant had no stimulatory effect with linearized plasmid DNA consistent with the low, near background level of activation observed on the other DNA substrates. To more closely interrogate how the extreme C-terminus is impacted by length we analyzed a series of DNA substrates with identical blunt end termini and of differing lengths. The results demonstrated that at 500bp in length, a modest but statistically significant decrease in activation is observed upon depleting the extreme C-terminus. Consistent with the 30, 60 and 400bp substrates, no effect of deleting the extreme-C-terminus was revealed on a 300bp substrate (S2 Fig). Considering the interesting effect of kinase activation as a function of overhangs and previous research, including our own, that has demonstrated the influence of regions of micro-homology within DNA termini on NHEJ catalyzed repair [2,29,30], we sought to determine if the different Ku constructs altered kinase activation as a function of complementary and non-complementary DNA termini. We observed no detectible differences in overall activation or Ku80/DNA-PKcs activation relationships on complementary versus non-complementary DNA termini (data not shown).

Overall, the data suggest that the Ku80/DNA-PKcs interactions necessary for DNA-PK activation cannot be defined to any one structural region of the Ku80 C-terminus regardless of DNA substrate. Instead different structural regions are required for kinase stimulation depending on the structure and length of the DNA substrate to which the complex is bound. Further, all previous models of Ku dependent activation of DNA-PK, including our own, have involved Ku remaining at DNA termini without regard to distant DNA length. These data show that Ku discriminates the length of the DNA cofactor to which it binds and activates DNA-PK accordingly through its C-terminus. A summary of the stimulatory effects of each of the structural Ku80 mutants normalized to wild type is shown in Table 1 and highlights the differences in activation observed.

### Terminal Pyrimidines Preferentially Stimulate DNA-PK

**Fig 6:**
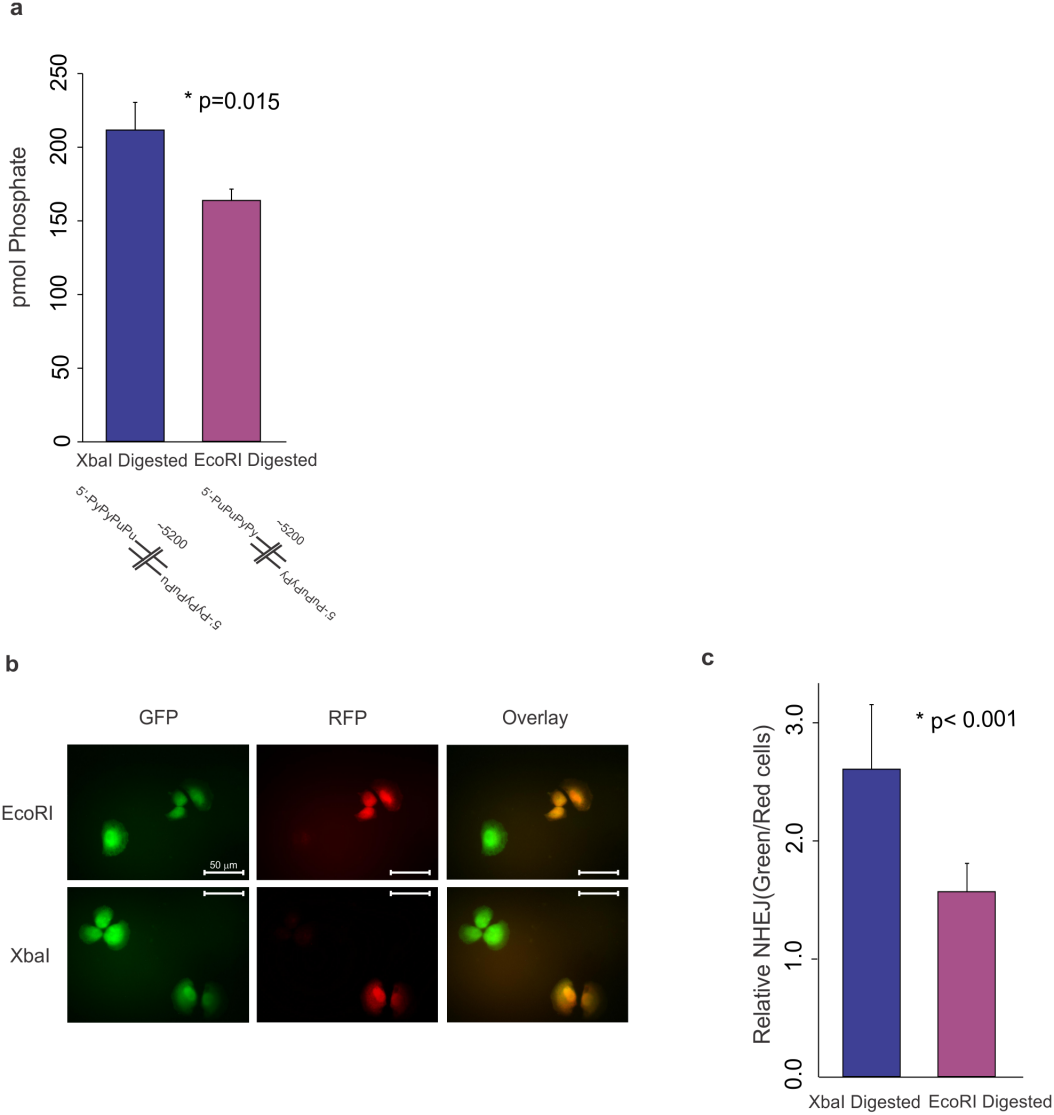
Preferential DNA-PK Activation by Terminal Pyrimidines Leads to Increased NHEJ. a) DNA-PK kinase activity is determined with wild type Ku and linearized reporter plasmid digested with restriction enzymes XbaI and EcoRI. DNA termini generated by digestion are depicted below the graph indicating locations of pyrimidines (Py) and purines (Pu). Activity is reported as pmol of phosphate transferred. b) Representative images analyzed for host cell reactivation assay showing GFP and RFP expression. Images were obtained using a 40x objective. Scale bar = 50 μm. c) Quantified results from host cell reactivation assay. Results are reported as ratio of green cells to red fluorescent cells relative to NHEJ activity. Results from XbaI digested DNA are shown in blue and results from EcoRI digested DNA are shown in purple. Data is presented as the mean and SD with asterisks indicating statistically significant differences.

The demonstration that DNA-PK is differentially activated as a function of DNA length and structure is reminiscent of our previous data demonstrating that a strand and sequence bias for activation [2,9]. Substrates with 5’ overhangs are of particular interest as the influence of the C-terminal domains on DNA-PKcs stimulation varied dramatically. In order to determine if overhang sequences influences the ability of the Ku80 C-terminal structural regions to stimulate the kinase, the same plasmid was digested with different sequence-specific restriction endonucleases. Despite the differences in terminal sequences, the influence of C-terminus was similar when both experiments when normalized to wild type (S3 Fig). Under both conditions the helical bundle was required for maximum stimulation while the core DNA binding/Ku70 dimerization domain and disordered linker regions both contributed to stimulation above the no Ku control. While the relationships between the abilities of the mutants remains the same (S3 Fig), the overall kinase activation from XhoI digested DNA was drastically greater than the activation observed by the BamHI digested DNA (Fig 5b). Despite the substantial differences in kinase stimulation, the DNA sequence differences in the ends generated from digestion with these restriction enzymes are subtle. While digestion from both enzymes results in a 4 base 5’ overhang, digestion by XhoI generates a 5’TCGA overhang and digestion by BamHI generates a 5’GATC overhang. Using additional restriction enzymes and a GFP reporter plasmid, we next tested whether the terminal pyrimidines generated by XhoI digestion were responsible for increased activation. XbaI digestion yields a 5’CTAG single stranded overhang while EcoRI digestion yields a 5‘AATT single stranded overhang. Consistent with our prediction, the linearized plasmid with terminal pyrimidines (XbaI digested) resulted in greater DNA-PK activation than that of terminal purines (EcoR1) (Fig 6a, S3 Table). Interestingly, both BamHI and EcoRI digested DNA contain single stranded overhangs with pyrimidines adjacent to the terminal purines however these ends do not stimulate DNA-PK as high as those with terminal pyrimidines. All four of the 5’ overhang-generating restriction enzymes used in this study result in single strand regions containing 2 pyrimidines and 2 purines and yet these cause drastically different levels of DNA-PK stimulation. This illustrates that subtle variations in DNA sequence can have profound effects on DNA-PK activation.

### Preferential DNA-PK Activation by Terminal Pyrimidines Leads to Increased NHEJ

Because DNA-PK activation is a major regulator in NHEJ [8], we asked if the sequence-specific activation by terminal pyrimidines would impact DNA DSB repair via NHEJ. To investigate this question, a host cell reactivation assay was employed utilizing a linearized plasmid encoding a green fluorescent protein (GFP) reporter gene that is expressed following NHEJ. This plasmid was linearized with either XbaI or EcoRI, both of which generate 4 base 5’ overhangs between the promoter and GFP expression reporter gene, however XbaI digestion yields an NHEJ substrate with terminal pyrimidines while EcoRI digestion yields an NHEJ substrate with terminal purines. Differences in transfection efficiency were controlled for by co-transfection with circular pCAG-dsRED which produces a red fluorescent protein (RFP) independent of NHEJ [22]. The results presented in Fig 6b are representative images of individual fields and an overlay of the images. Quantified results are presented in Fig 6c as a ratio of green cells: red cells and show that NHEJ substrates generated with XbaI digestion are repaired more efficiently than those generated via EcoRI digestion (p < 0.001). We have previously shown with this plasmid that expression of the GFP is inhibited by the DNA-PK inhibitor, NU7441, and absent in NHEJ deficient cells [22]. Thus expression of GFP is indicative of NHEJ and it is clear that NHEJ substrates with terminal pyrimidines are repaired more efficiently than those of with terminal purines. These results are consistent with a model where the sequences at the termini of DSBs influence DNA-PK activation and ultimately DSB repair via NHEJ.

## Discussion

Overall, our results establish distinct mechanisms by which DNA-PK is able to respond to a variety of DNA substrates to support repair. We show that the structures of individual DNA substrates dictate specific structure/function relationships between the Ku80 C-terminus and DNA-PKcs. These relationships are complex and are intimately tied to the protein structure and the structure of the DNA substrate to which the holoenzyme is bound. Previous studies have either concluded that the extreme C-terminus is critical for kinase activity or that the C-terminus is entirely dispensable for activation [10,11]. Results presented here indicate that each structural region within the C-terminus of Ku80 is distinctly necessary for stimulation of the kinase depending on the structure of the DNA substrate (Table 1). We have observed that multiple structural features of the DNA influence Ku80/DNA-PKcs interactions and DNA-PK activation including: 1) length of DNA 2) presence of overhangs 3) orientation of overhangs and 4) sequence of overhangs. Thus differing conclusions from previous studies are likely due to differences in methodology with regard to the structure of the DNA substrates used to evaluate DNA-PK activation. Further, our *in vitro* data assessing the effect of these structural regions on DNA-PKcs-DNA binding activity suggests that differences in DNA-PK activation are a function of reduced catalytic activity and not substrate binding activity as measured by DNA-PKcs recruitment to DNA (Fig 4). In addition some substrates promote complex formation more than others, i.e. 60bp substrates, independent of the Ku80 C-terminus (Fig 4). This is consistent with previous reports that the Ku80 C-terminus is dispensable for DNA-PKcs recruitment to DSBs *in vivo* [11].

**Fig 7:**
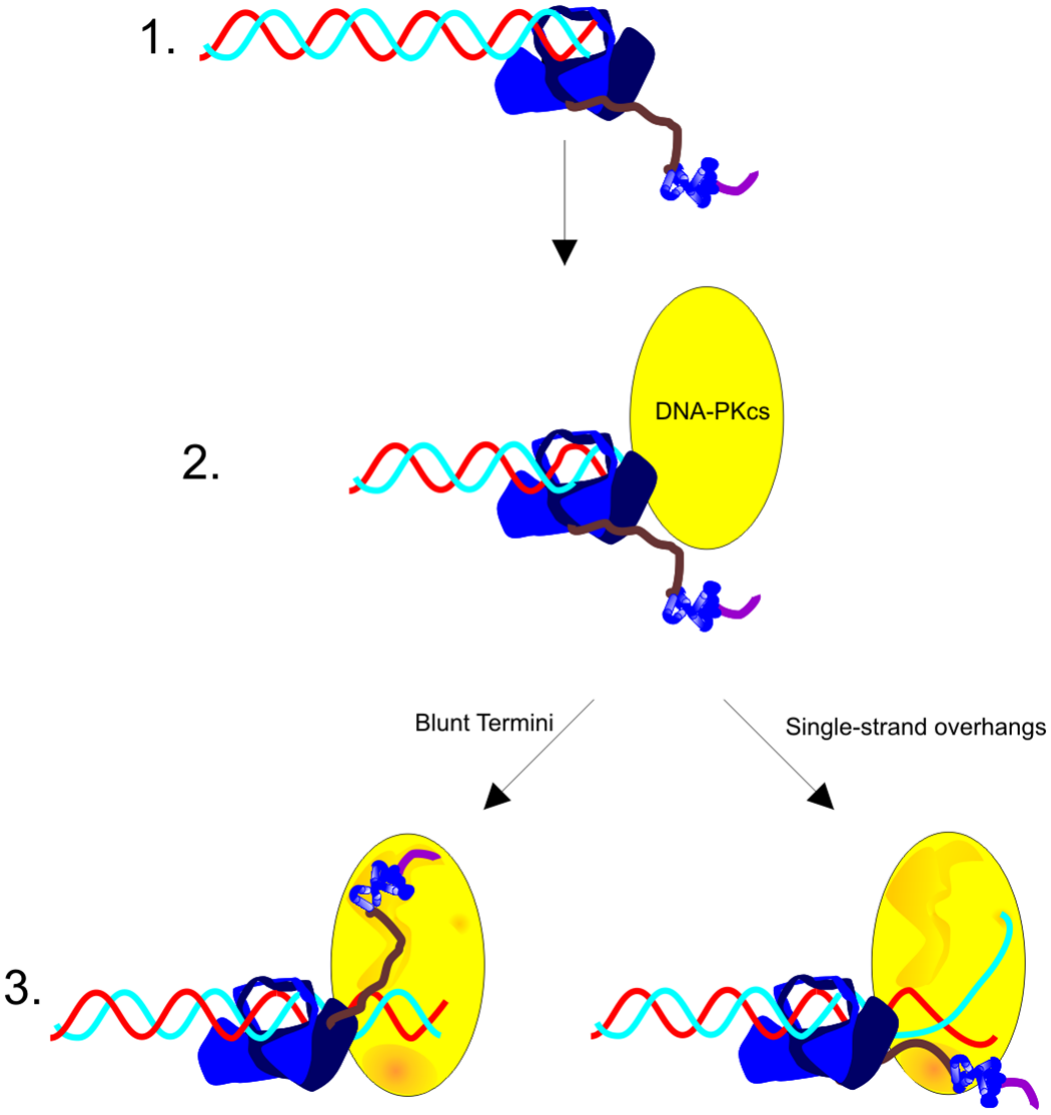
Model of Distinct Mechanisms of DNA-PK Activation Dependent on DNA Cofactor Structure. 1. The Ku heterodimer binds to DNA termini and undergoes a conformational change involving the C-terminus of Ku80 [14]. 2. DNA-PKcs is recruited to the DNA terminus through interactions with Ku but independent of interactions with the Ku80 C-terminus (See Fig 3). 3. When bound to blunt-ended DNA, DNA-PKcs is activated through interactions with the disordered linker, helical bundle, and extreme C-terminus of the Ku80 C-terminus. When bound to DNA with 3’ or 5’ overhangs, DNA-PKcs is activated through interactions with the disordered linker and helical bundle of the Ku80 C-terminus. The 5’ end of DNA activates the kinase while the 3’ end is free to anneal across the synapse [2]. A DSB with a 5’ overhang is depicted. DNA sequence specific interactions between the 5’ overhang and DNA-PKcs are indicated in the model.

The sheer size of the DNA-PK complex has hindered high resolution structural analysis. DNA-PKcs is approximately 470kDa and when in complex with the 150kDa Ku heterodimer, is ~620kDa. Keeping in mind that DNA-PK complexes exist on both sides of the DSB, the effective size of the active complex as it performs its function in NHEJ is over 1 mega Da. Despite this, a crystal structure of DNA-PKcs has been solved to 6.6 Å [31]. Importantly, crystals only formed in the presence of the Ku80 C-terminus. While the site(s) of Ku80 C-terminus/DNA-PKcs interactions could not be discerned from these studies, the authors were able to conclude that such interactions are necessary for stabilizing highly mobile regions of DNA-PKcs. This stabilization effect on mobile regions support an interaction but certainly does not limit these complex interactions to the region being stabilized. More recently small angle X-ray scattering data confirmed our initial finding that the C-terminus experiences a conformational change upon Ku binding to DNA and becomes more accessible potentially to allow for additional protein interactions [15]. Further they show that different surfaces of DNA-PKcs interact across the synapse when the complexes are bound to different DNA substrates. This is consistent with our data and suggests distinct protein/protein interactions are necessary for activation on different DNA substrates. These studies together with our enzymology data support a model by which DNA-PK responds to structurally distinct DSBs by “sensing” these structures through its interactions with Ku80 (Fig 7). On substrates with 5’ or 3’ overhangs, the helical bundle and linker regions of Ku80 stimulate kinase activity. The overhangs themselves play unique roles in this process as terminal pyridines preferentially stimulate kinase activity while 3’ overhangs align DNA termini across the synapse [2]. Interestingly, blunt-ended substrates uniquely require the extreme C-terminus for maximum activation. DSBs with blunt-ended termini arise in the cell from replication through nicked DNA. Perhaps these events which produce one-sided DSBs are “sensed” through interactions with the extreme C-terminus of Ku80. Previous reports indicate that extended conformations of the C-terminus are capable of interactions with DNA-PKcs across the synapse [15]. Thus the stimulatory interactions reported here may occur either in-*cis* or in*-trans*.

Here we present the novel finding that Ku discriminate terminal structures of DNA substrates and activates DNA-PKcs through distinct mechanisms. The ability of Ku to distinguish terminal DNA structures is in accord with its terminal DNA binding activity [18]. Interestingly the C-terminus of Ku80 is dispensable for activation of 30bp Y substrates as is Ku in its entirety for 60bp Y substrates (Fig 2). DNA strand separation being a likely component of the DNA-PK activation model is consistent with the structural studies of DNA-PKcs which reveal a punitive ssDNA binding site along with a dsDNA binding site [15,32]. Further our previously published results reveal unique contributions from single strand overhangs implicate strand separation as a likely intermediate during NHEJ [8].

An interesting and unexpected finding from this work is that Ku also utilizes distinct mechanisms of DNA-PKcs activation based on DNA length. Previous studies which directly visualized Ku/DNA interactions reveal that very few Ku molecules translocate internally on the DNA substrate with the vast majority remaining at the termini [27,33]. Structural studies reveal that Ku directly binds approximately 20bp or 2 DNA turns [18]. Despite this, Ku distinguishes between 400bp and 500bp DNA substrates as evidenced by the distinct mechanisms utilized to activate DNA-PKcs (S3 Fig). How this distinction occurs remains unknown but suggests that Ku/DNA interactions are more complex than previously thought.

To the best of our knowledge, we provide the first evidence that where a DSB occurs may influence how efficiently it can be repaired as a function of DNA-PK activation. Indeed, DNA-PK displays a dramatic sequence bias for terminal pyrimidines even over sequences with terminal purines containing adjacent pyrimidines. We did not observe any differences DNA-PKcs stimulation by the Ku truncation constructs due to terminal sequence variations. Clearly when it comes to DNA-PK activation, not all bases are created equal and their precise location at the DSB has an impact on repair. The mechanisms by which terminal pyrimidines are more stimulatory than terminal purines is still unknown, but are possibly due to direct ssDNA interactions with DNA-PKcs [15]. The fact that Ku is not involved in sequence discrimination is consistent with structural data which shows that Ku interacts exclusively with the phosphodiester backbone of DNA. Further recent high profile work indicates that Ku is a 5’-dRP/AP lyase [34,35] and excises nucleotide damage near broken ends of DNA. These reports show that Ku cannot distinguish normal bases from abasic sites on internal sequences but can accomplish this at DNA termini. These data are consistent with our finding that base distinction by this complex occurs only at DNA termini. Therefore Ku’s inability to differentiate sequence variation is not surprising. Structural studies of DNA-PKcs, on the other hand, reveal two potential DNA binding domains [15,32]. The larger of the two located in the palm domain is almost assuredly involved in binding dsDNA while the smaller domain located in the head domain has been implicated in binding ssDNA [32]. Perhaps the single ring structure of pyrimidines interacts more strongly with this putative ssDNA binding domain as compared to the double ring structure of purines. This mechanism is currently under investigation. Further, there are numerous other DNA structures and conformations that exist throughout the genome, all of which are susceptible to DSBs. It will be important to evaluate how these regions influence DNA-PK activation in light of the differential activation and repair observed in this study.

## Supporting Information

S1 Fig DNA and Ku Titrations Used to Determine Optimal Conditions of Kinase Assays. a) Kinase Assays were performed with 30bp and indicated amounts of DNA. These data were used to determine that 500fmol of DNA results in excess of DNA cofactor. b) Kinase Assays were performed with indicated amounts of Wild type Ku. 50 Units was found to be optimal for kinase assays and Ku was found to be in excess. Kinase activity is reported the mean and SD of pmol of phosphate transferred.
(PDF)

S2 Fig Kinase Assays Using Blunt End Substrates. a) 500bp Blunt End Substrates require the extreme C-terminal region of Ku80 for maximum activation. All differences between groups are statistically significant. b) 300bp Blunt End Substrates do not require the extreme C-terminal region of Ku80 for maximum activation. Differences between wild type and C-L-H are not statistically significant. Kinase activity is reported as the mean and SD of pmol of phosphate transferred.
(PDF)

S3 Fig Distinct Influences of the Ku80 C-terminus on DNA-PK Activation with Linearized Plasmid DNA. DNA-PK kinase stimulation with plasmid DNA linearized with XhoI and BamHI generating 4 base 5’ single stranded overhangs. DNA substrates are depicted pictorially with the terminal bases generated by digestion depicted below each graph indicating locations of pyrimidines (Py) and purines (Pu). The data from Fig 5b was used to calculate the kinase activity as reported as percentage of wild type activity for each DNA. The mean and SD are presented and asterisks indicate statistically significant differences compared to wild type (p <0.05).
(PDF)

S1 Table Oligonucleotide DNA substrates. (PDF)

S2 Table 400 bp DNA substrate sequences. (PDF)

S3 Table Restriction enzyme recognition sequences. (PDF)

## References

[1] Drouet J, Frit P, Delteil C, de Villartay JP, Salles B, Calsou P (2006) Interplay between Ku, artemis, and the DNA-dependent protein kinase catalytic subunit at DNA ends. J Biol Chem 281: 27784–27793. 16857680 10.1074/jbc.M603047200

[2] Pawelczak KS, Turchi JJ (2008) A mechanism for DNA-PK activation requiring unique contributions from each strand of a DNA terminus and implications for microhomology-mediated nonhomologous DNA end joining. Nucleic Acids Res 36: 4022–4031. 10.1093/nar/gkn344 18515838 PMC2475626

[3] Furuta T, Redon C, Pilch D, Sedelnikova O, Kohlhagen G, Kirchgessner CU, et al (2002) ATR- and DNA-PK- dependent phosphorylation of histone H2AX by replication-mediated DNA double-strand break induced by camptothecin. Gastroenterology 122: S918.

[4] Reitsema T, Klokov D, Banath JP, Olive PL (2005) DNA-PK is responsible for enhanced phosphorylation of histone H2AX under hypertonic conditions. Dna Repair 4: 1172–1181. 16046194 10.1016/j.dnarep.2005.06.005

[5] Nuss JE, Patrick SM, Oakley GG, Alter GM, Robison JG, Dixon K, et al (2005) DNA Damage Induced Hyperphosphorylation of Replication Protein A. 1. Identification of novel sites of phosphorylation in response to DNA damage. Biochemistry 44: 8428–8437. 15938632 10.1021/bi0480584 PMC4331072

[6] Liu S, Opiyo SO, Manthey K, Glanzer JG, Ashley AK, Amerin C, et al (2012) Distinct roles for DNA-PK, ATM and ATR in RPA phosphorylation and checkpoint activation in response to replication stress. Nucleic Acids Res 40: 10780–10794. gks849 [pii]; 10.1093/nar/gks849 [doi]. 22977173 PMC3510507

[7] Lees-Miller SP, Chen YR, Anderson CW (1990) Human cells contain a DNA-activated protein kinase that phosphorylates simian virus 40 T antigen, mouse p53, and the human Ku autoantigen. Mol Cell Biol 10: 6472–6481. 2247067 10.1128/mcb.10.12.6472-6481.1990 PMC362924

[8] Woods D, Turchi JJ (2013) Chemotherapy induced DNA damage response: Convergence of drugs and pathways. Cancer Biol Ther 14: 379–389. 23761 [pii]. 10.4161/cbt.23761 23380594 PMC3672181

[9] Pawelczak KS, Andrews BJ, Turchi JJ (2005) Differential activation of DNA-PK based on DNA strand orientation and sequence bias. Nucleic Acids Res 33: 152–161. 15640450 10.1093/nar/gki157 PMC546145

[10] Falck J, Coates J, Jackson SP (2005) Conserved modes of recruitment of ATM, ATR and DNA-PKcs to sites of DNA damage. Nature 434: 605–611. 15758953 10.1038/nature03442

[11] Weterings E, Verkaik NS, Keijzers G, Florea BI, Wang SY, Ortega LG, et al (2009) The Ku80 carboxy terminus stimulates joining and artemis-mediated processing of DNA ends. Mol Cell Biol 29: 1134–1142. 10.1128/MCB.00971-08 19103741 PMC2643835

[12] Gell D, Jackson SP (1999) Mapping of protein-protein interactions within the DNA-dependent protein kinase complex. Nucleic Acids Res 27: 3494–3502. 10446239 10.1093/nar/27.17.3494 PMC148593

[13] Harris R, Esposito D, Sankar A, Maman JD, Hinks JA, Pearl LH, Driscoll PC (2004) The 3D solution structure of the C-terminal region of Ku86 (Ku86CTR). J Mol Biol 335: 573–582. 14672664 10.1016/j.jmb.2003.10.047

[14] Lehman JA, Hoelz DJ, Turchi JJ (2008) DNA-Dependent Conformational Changes in the Ku Heterodimer. Biochemistry 47: 4359–4368. 10.1021/bi702284c 18355052 PMC2432109

[15] Hammel M, Yu Y, Mahaney BL, Cai B, Ye R, Phipps BM, Rambo RP, et al (2010) Ku and DNA-dependent protein kinase dynamic conformations and assembly regulate DNA binding and the initial non-homologous end joining complex. J Biol Chem 285: 1414–1423. 10.1074/jbc.M109.065615 19893054 PMC2801267

[16] Shibata A, Barton O, Noon AT, Dahm K, Deckbar D, Goodarzi AA, et al (2010) Role of ATM and the damage response mediator proteins 53BP1 and MDC1 in the maintenance of G(2)/M checkpoint arrest. Mol Cell Biol 30: 3371–3383. 10.1128/MCB.01644-09 20421415 PMC2897583

[17] Bennett SM, Woods DS, Pawelczak KS, Turchi JJ (2012) Multiple protein-protein interactions within the DNA-PK complex are mediated by the C-terminus of Ku 80. Int J Biochem Mol Biol 3: 36–45. 22509479 PMC3325771

[18] Walker JR, Corpina RA, Goldberg J (2001) Structure of the Ku heterodimer bound to DNA and its implications for double-strand break repair. Nature 412: 607–614. 11493912 10.1038/35088000

[19] Hermanson IL, Turchi JJ (2000) Overexpression and purification of human XPA using a Baculovirus expression system. Protein Expression and Purification 19: 1–11. 10833384 10.1006/prep.2000.1224

[20] Earley JN, Turchi JJ (2011) Interrogation of nucleotide excision repair capacity: impact on platinum-based cancer therapy. Antioxid Redox Signal 14: 2465–2477. 10.1089/ars.2010.3369 20812782 PMC3096502

[21] Andrews BJ, Lehman JA, Turchi JJ (2006) Kinetic analysis of the Ku-DNA binding activity reveals a redox-dependent alteration in protein structure that stimulates dissociation of the Ku-DNA complex. J Biol Chem 281: 13596–13603. 16537541 10.1074/jbc.M512787200 PMC2432111

[22] Sears CR, Turchi JJ (2012) Complex Cisplatin-Double Strand Break (DSB) Lesions Directly Impair Cellular Non-Homologous End-Joining (NHEJ) Independent of Downstream Damage Response (DDR) Pathways. J Biol Chem 287: 24263–24272. M112.344911 [pii]; 10.1074/jbc.M112.344911 [doi]. 22621925 PMC3397852

[23] Singleton BK, Torres-Arzayus MI, Rottinghaus ST, Taccioli GE, Jeggo PA (1999) The C terminus of Ku80 activates the DNA-dependent protein kinase catalytic subunit. Mol Cell Biol 19: 3267–3277. 10207052 10.1128/mcb.19.5.3267 PMC84121

[24] Rivera-Calzada A, Spagnolo L, Pearl LH, Llorca O (2007) Structural model of full-length human Ku70-Ku80 heterodimer and its recognition of DNA and DNA-PKcs. EMBO Rep 8: 56–62. 17159921 10.1038/sj.embor.7400847 PMC1796749

[25] Spagnolo L, Rivera-Calzada A, Pearl LH, Llorca O (2006) Three-dimensional structure of the human DNA-PKcs/Ku70/Ku80 complex assembled on DNA and its implications for DNA DSB repair. Mol Cell 22: 511–519. 16713581 10.1016/j.molcel.2006.04.013

[26] Ma Y, Lieber MR (2001) DNA length-dependent cooperative interactions in the binding of Ku to DNA. Biochemistry 40: 9638–9646. 11583164 10.1021/bi010932v

[27] Pang D, Yoo S, Dynan WS, Jung M, Dritschilo A (1997) Ku proteins join DNA fragments as shown by atomic force microscopy. Cancer Res 57: 1412–1415. 9108436

[28] Jackson SP, Bartek J (2009) The DNA-damage response in human biology and disease. Nature 461: 1071–1078. nature08467 [pii]; 10.1038/nature08467 [doi]. 19847258 PMC2906700

[29] Willers H, Husson J, Lee LW, Hubbe P, Gazemeier F, Powell SN, et al (2006) Distinct mechanisms of nonhomologous end joining in the repair of site-directed chromosomal breaks with noncomplementary and complementary ends. Radiat Res 166: 567–574. 17007549 10.1667/RR0524.1

[30] Katsura Y, Sasaki S, Sato M, Yamaoka K, Suzukawa K, Nagasawa T, et al (2007) Involvement of Ku80 in microhomology-mediated end joining for DNA double-strand breaks *in vivo* . DNA Repair (Amst) 6: 639–648. 17236818 10.1016/j.dnarep.2006.12.002

[31] Sibanda BL, Chirgadze DY, Blundell TL (2010) Crystal structure of DNA-PKcs reveals a large open-ring cradle comprised of HEAT repeats. Nature 463: 118–121. 10.1038/nature08648 20023628 PMC2811870

[32] Williams DR, Lee KJ, Shi J, Chen DJ, Stewart PL (2008) Cryo-EM structure of the DNA-dependent protein kinase catalytic subunit at subnanometer resolution reveals alpha helices and insight into DNA binding. Structure 16: 468–477. 10.1016/j.str.2007.12.014 18334221 PMC2323513

[33] Cary RB, Peterson SR, Wang J, Bear DG, Bradbury EM, Chen DJ (1997) DNA looping by Ku and the DNA-dependent protein kinase. Proc Natl Acad Sci USA 94: 4267–4272. 9113978 10.1073/pnas.94.9.4267 PMC20711

[34] Roberts SA, Strande N, Burkhalter MD, Strom C, Havener JM, Hasty P, et al (2010) Ku is a 5'-dRP/AP lyase that excises nucleotide damage near broken ends. Nature 464: 1214–1217. 10.1038/nature08926 20383123 PMC2859099

[35] Strande N, Roberts SA, Oh S, Hendrickson EA, Ramsden DA (2012) Specificity of the dRP/AP lyase of Ku promotes nonhomologous end joining (NHEJ) fidelity at damaged ends. J Biol Chem 287: 13686–13693. M111.329730 [pii]; 10.1074/jbc.M111.329730 [doi]. 22362780 PMC3340204

