# Supplementary figures and images for: Recognition of DNA Termini by the C-Terminal Region of the Ku80 and the DNA-Dependent Protein Kinase Catalytic Subunit
Source: PLoS One. 2015 May 15;10(5):e0127321. doi: 10.1371/journal.pone.0127321 (PMC4433226; doi:10.1371/journal.pone.0127321)

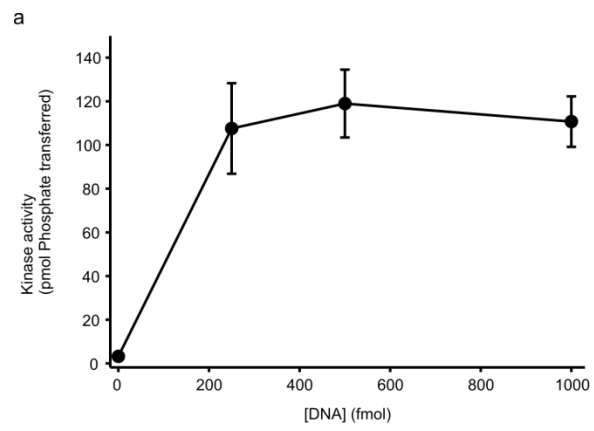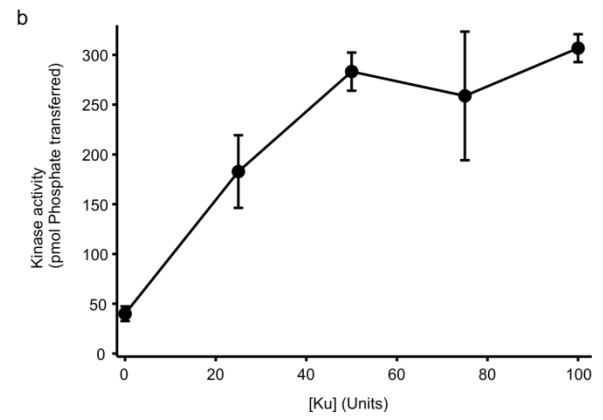

**S1 Fig**

Supplement: S1 Fig — a) Kinase Assays were performed with 30bp and indicated amounts of DNA. These data were used to determine that 500fmol of DNA results in excess of DNA cofactor. b) Kinase Assays were performed with indicated amounts of Wild type Ku. 50 Units was found to be optimal for kinase assays and Ku was found to be in excess. Kinase activity is reported the mean and SD of pmol of phosphate transferred. (PDF) [file pone.0127321.s001.pdf]

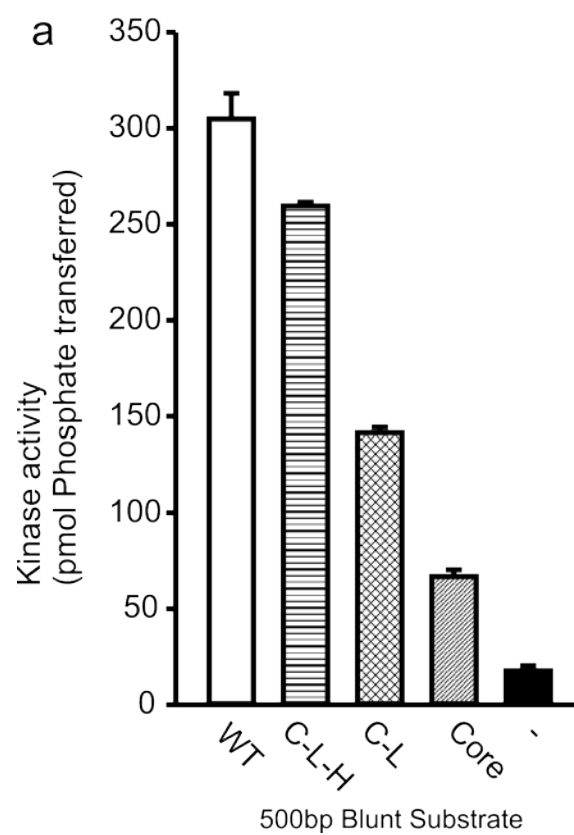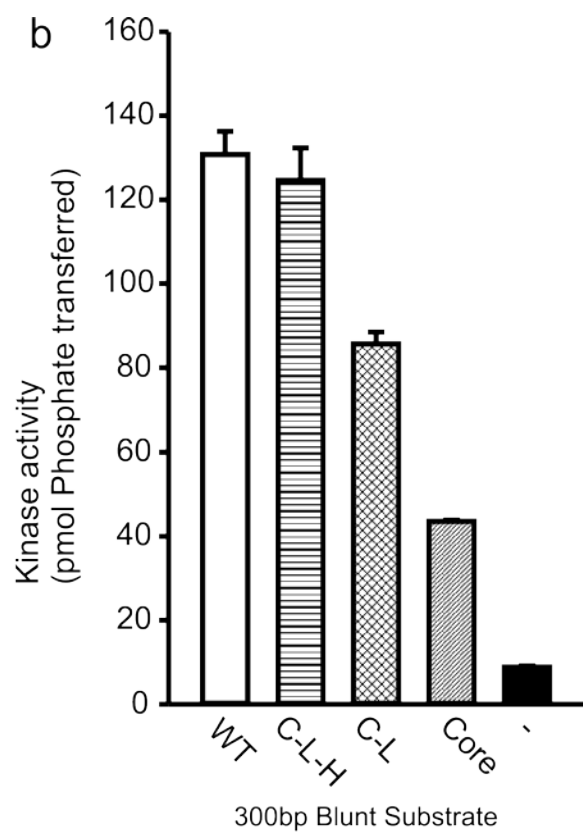

**S2 Fig**

Supplement: S2 Fig — a) 500bp Blunt End Substrates require the extreme C-terminal region of Ku80 for maximum activation. All differences between groups are statistically significant. b) 300bp Blunt End Substrates do not require the extreme C-terminal region of Ku80 for maximum activation. Differences between wild type and C-L-H are not statistically significant. Kinase activity is reported as the mean and SD of pmol of phosphate transferred. (PDF) [file pone.0127321.s002.pdf]

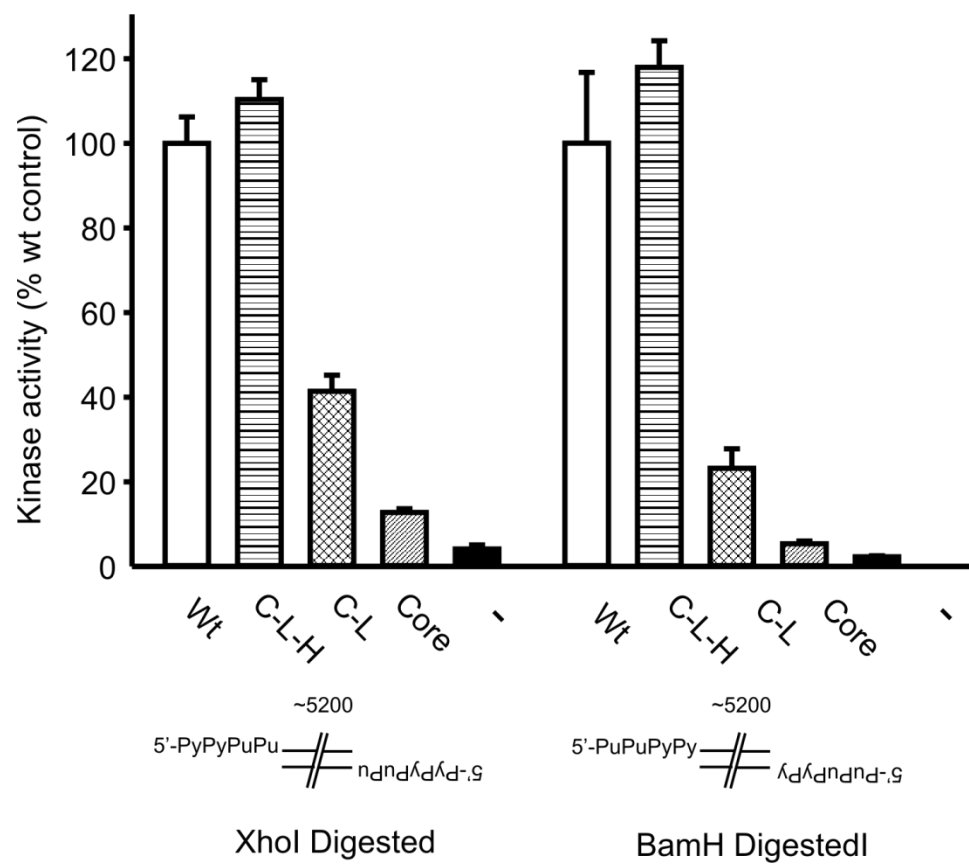

**S3 Fig**

Supplement: S3 Fig — DNA-PK kinase stimulation with plasmid DNA linearized with XhoI and BamHI generating 4 base 5’ single stranded overhangs. DNA substrates are depicted pictorially with the terminal bases generated by digestion depicted below each graph indicating locations of pyrimidines (Py) and purines (Pu). The data from Fig 5b was used to calculate the kinase activity as reported as percentage of wild type activity for each DNA. The mean and SD are presented and asterisks indicate statistically significant differences compared to wild type (p <0.05). (PDF) [file pone.0127321.s003.pdf]
