# Supplementary material for: Recognition of DNA Termini by the C-Terminal Region of the Ku80 and the DNA-Dependent Protein Kinase Catalytic Subunit
Source: PLoS One. 2015 May 15;10(5):e0127321. doi: 10.1371/journal.pone.0127321 (PMC4433226; doi:10.1371/journal.pone.0127321)
Supplement: S1 Table — (PDF) [file pone.0127321.s004.pdf]

S1 Table

Oligonucleotide DNA Substrates

Name	Annealed with	Overhangs	Sequence (5'-3')
30mer Top	30mer Bottom	None	CTGAAGGCAGTGTCACCTCTGTTGGAAGTG
30mer Bottom	30mer Top	None	CACTTCCAACAGAGGTGACACTGCCTTCAG
30mer 3' Top	30mer 3' Bottom	4 base 3'	CTGAAGGCAGTGTCACCTCTGTTGGAAGTGTGCA
30mer 3' Bottom	30mer 3' Top	4 base 3'	CACTTCCAACAGAGGTGACACTGCCTTCAGTGCA
30mer 5' Top	30mer 5' Bottom	4 Base 5'	TGCACTGAAGGCAGTGTCACCTCTGTTGGAAGTG
30mer 5' Bottom	30mer 5' Top	4 Base 5'	TGCACACTTCCAACAGAGGTGACACTGCCTTCAG
60mer Top	60mer Bottom	None	GTTAAGTATCTGCATCTTACTTGACGCATGCAATCGTC ACGTGCTAGACTACTGGTCAAG
60mer Bottom	60mer Top	None	CTTGACCAGTAGTCTAGCACGTGACGATTGCATGCGTC AAGTAAGATGCAGATACTTAAC
60mer 3' Top	60mer 3' Bottom	4 Base 3'	TGCAGTTAAGTATCTGCATCTTACTTGACGCATGCAAT CGTCACGTGCTAGACTACTGGTCAAG
60mer 3' Bottom	60mer 3' Top	4 Base 3'	ACCAGTAGTCTAGCACGTGACGATTGCATGCGTCAAG TAAGATGCAGATACTTAACTGCATGCA
60mer 5' Top	60mer 5' Bottom	4 Base 5'	TGCAGTTAAGTATCTGCATCTTACTTGACGCATGCAAT CGTCACGTGCTAGACTACTGGTCAAG
60mer 5' Bottom	60mer 5' Top	4 Base 5'	TGCACTTGACCAGTAGTCTAGCACGTGACGATTGCATG CGTCAAGTAAGATGCAGATACTTAAC
