# Supplementary material for: Recognition of DNA Termini by the C-Terminal Region of the Ku80 and the DNA-Dependent Protein Kinase Catalytic Subunit
Source: PLoS One. 2015 May 15;10(5):e0127321. doi: 10.1371/journal.pone.0127321 (PMC4433226; doi:10.1371/journal.pone.0127321)
Supplement: S2 Table — (PDF) [file pone.0127321.s005.pdf]

S2 Table

~400 base pair DNA Substrate Sequences.

Name	Length (bp)	Overhangs	Sequence (5'-3')
Blunt-ended gBlock	421	None	TGA CAT TGA ATT CTC TCC ACC ATA TGG AAT TTG ATT ATG TAA TAT GCG AAG AAT GTG GGA AAG AAT TTA TGG ATT CTT ATC TTA TGA ACC ACT TTG ATT TGC CAA CTT GTG ATA ACT GCA GAG ATG CTG ATG ATA AAC ACA AGC TTA TAA CCA AAA CAG AGG CAA AAC AAG AAT ATC TTC TGA AAG ACT GTG ATT TAG AAA AAA GAG AGC CAC CTC TTA AAT TTA TTG TGA AGA AGA ATC CAC ATC ATT CAC AAT GGG GTG ATA TGA AAC TCT ACT TAA AGT TAC AGA TTG TGA AGA GGT CTC TTG AAG TTT GGG GTA GTC AAG AAG CAT TAG AAG AAG CAA AGG AAG TCC GAC AGG AAA ACC GAG AAA AAA TGA AAC AGA AGA AAT TTT AGT AGG GAT CCG TGG ATT G AA TTC CCT CCG A
KpnI gBlock	397	4 base 3'	CTT TCC ACC ATA TGG AAT TTG ATT ATG TAA TAT GCG AAG AAT GTG GGA AAG AAT TTA TGG ATT CTT ATC TTA TGA ACC ACT TTG ATT TGC CAA CTT GTG ATA ACT GCA GAG ATG CTG ATGATA AAC ACA AGC TTA TAA CCA AAA CAG AGG CAA AAC AAG AAT ATC TTC TGA AAG ACT GTG ATT TAG AAA AAA GAG AGC CAC CTC TTA AAT TTA TTG TGA AGA AGA ATC CAC ATC ATT CAC AAT GGG GTG ATA TGA AAC TCT ACT TAA AGT TAC AGA TTG TGA AGA GGT CTC TTG AAG TTT GGG GTA GTC AAG AAG CAT TAG AAG AAG CAA AGG AAG TCC GAC AGG AAA ACC GAG AAA AAA TGA AAC AGA AGA AAT TTT AGT AGG GAT CCG TGG ACC GGT AC
EcoRI gBlock	397	4 base 5'	AATT CTC TCC ACC ATA TGG AAT TTG ATT ATG TAA TAT GCG AAG AAT GTG GGA AAG AAT TTA TGG ATT CTT ATC TTA TGA ACC ACT TTG ATT TGC CAA CTT GTG ATA ACT GCA GAG ATG CTG ATG ATA AAC ACA AGC TTA TAA CCA AAA CAG AGG CAA AAC AAG AAT ATC TTC TGA AAG ACT GTG ATT TAG AAA AAA GAG AGC CAC CTC TTA AAT TTA TTG TGA AGA AGA ATC CAC ATC ATT CAC AAT GGG GTG ATA TGA AAC TCT ACT TAA AGT TAC AGA TTG TGA AGA GGT CTC TTG AAG TTT GGG GTA GTC AAG AAG CAT TAG AAG AAG CAA AGG AAG TCC GAC AGG AAA ACC GAG AAA AAA TGA AAC AGA AGA AAT TTT AGT AGG GAT CCG TGG ATT G
