# Supplementary material for: Recognition of DNA Termini by the C-Terminal Region of the Ku80 and the DNA-Dependent Protein Kinase Catalytic Subunit
Source: PLoS One. 2015 May 15;10(5):e0127321. doi: 10.1371/journal.pone.0127321 (PMC4433226; doi:10.1371/journal.pone.0127321)
Supplement: S3 Table — (PDF) [file pone.0127321.s006.pdf]

S3 Table

Sequences Recognized by Restriction Enzymes Used

Enzyme	Sequence and Site of Cuts
EcoRV	5′. . . G A T A T C . . . 3′<br>3′. . . C T A T A G . . . 5′
KpnI	5′. . . G G T A C C . . . 3′<br>3′. . . C C A T G G . . . 5′
EcoRI	5′. . . G A A T T C . . . 3′<br>3′. . . C T T A A G . . . 5′
XhoI	5′. . . C T C G A G . . . 3′<br>3′. . . G A G C T C . . . 5′
BamHI	5′. . . G G A T C C . . . 3′<br>3′. . . C C T A G G . . . 5′
XbaI	5′. . . T C T A G A . . . 3′<br>3′. . . A G A T C T . . . 5′
